# Prevalence of pharmacologically treated attention deficit hyperactivity disorder in children, adolescents, and adults: systematic review and meta-analysis

**DOI:** 10.3389/fpsyt.2026.1854611

**Published:** 2026-07-10

**Authors:** Sara Popit, Spela Pfeifer, Igor Locatelli, Matej Stuhec

**Affiliations:** 1 University of Ljubljana, Faculty of Pharmacy, Ljubljana, Slovenia; 2 Pomurske Lekarne, Murska Sobota, Slovenia; 3 Department of Clinical Pharmacy & Pharmacology, Faculty of Medicine Maribor, University of Maribor, Maribor, Slovenia; 4 Department of Clinical Pharmacy, Ormoz Psychiatric Hospital, Ormoz, Slovenia

**Keywords:** adolescents and adults, attention deficit hyperactivity disorder, children, meta-analysis, pharmacological treatment, prevalence

## Abstract

**Introduction:**

Attention deficit hyperactivity disorder (ADHD) is one of the most common neurodevelopmental paediatric disorders and persists into adulthood, although it is frequently underdiagnosed and underrecognized in adult populations. In this context, the prevalence of pharmacologically treated individuals diagnosed with ADHD represents an important quality indicator for ADHD management.

**Aim:**

To estimate the pooled prevalence of pharmacologically treated individuals with ADHD in different age groups in Europe and worldwide.

**Methods:**

A comprehensive search of PubMed/MEDLINE was conducted to identify relevant articles published up to October 4, 2024. The present systematic review and meta-analysis examined ADHD prevalence using clinically confirmed diagnoses and treatment data from official records. The exclusion criteria included studies that lacked clinical confirmation of ADHD and/or relied exclusively on parental reports for diagnostic or medication information. The prevalence of pharmacologically treated individuals with ADHD was calculated as a percentage, with a 95% confidence interval (CI). A meta-analysis was performed in R using a random-effects model. Heterogeneity was calculated using I². Prediction intervals were additionally computed to reflect the expected range of prevalence in future studies. Risk of bias was assessed for all included studies using a standardized, previously published methodology. The study was prospectively registered in PROSPERO (CRD42020200220) and adhered to the PRISMA guidelines for systematic review and meta-analysis (2020).

**Results:**

The systematic review identified 13 studies (12 studies included in the meta-analysis) with substantial variation in age-specific reporting. The pooled prevalence of pharmacologically treated ADHD was 73.4% (95% CI: 63.4–81.5), with extremely high between-study heterogeneity and wide 95% prediction interval (29.6%–94.5%), reflecting substantial variation across settings. The pooled prevalence estimate should be interpreted with caution due to substantial between-study heterogeneity and is not intended for direct clinical inference. Geographic analyses revealed no significant variation across countries. Sex-stratified analyses showed no significant difference between males and females, although point estimates were slightly higher in males.

**Conclusion:**

The prevalence of pharmacological treatment among individuals with ADHD appears to vary across age groups and settings Overall, findings indicate substantial variation in pharmacological treatment of ADHD by age, with consistently high heterogeneity limiting the precision of pooled estimates.

**Systematic review registration:**

https://www.crd.york.ac.uk/PROSPERO/, identifier CRD42020200220.

## 1 Introduction

Attention deficit hyperactivity disorder (ADHD) is a neurodevelopmental disorder characterized by developmentally inappropriate levels of attention, hyperactivity, and/or impulsivity (1–3). It affects approximately 4–8% of children and adolescents and often persists into adulthood, with adult prevalence estimates 2–3% (1, 4–10). Although epidemiological studies indicate that ADHD prevalence is consistent worldwide when uniform diagnostic criteria are applied, administrative prevalence, reflecting clinical diagnosis rates, varies substantially both between and within countries (11).

Management of ADHD involves both pharmacological and non-pharmacological approaches (12–14). Pharmacological treatment is commonly recommended as a first-line treatment in many clinical guidelines, particularly for adults with ADHD (15). The medications used can be classified into stimulants (e.g., methylphenidate and amphetamines) and non-stimulants (e.g., atomoxetine) (16). Stimulants are considered first-line treatment due to their medium-to-high effect sizes and well-established efficacy (16). Non-stimulant medications are typically reserved for cases where stimulants are contraindicated, poorly tolerated, or ineffective (13). Non-pharmacological interventions are typically considered first-line treatments for children and adolescents with ADHD, while pharmacological therapy is generally preferred for adults (13, 15). ADHD is often managed with long-term pharmacological treatment, and many individuals remain on medication for several years (14). Current treatment guidelines recommend regular evaluations throughout treatment at least once a year (14, 15, 17). In this context, the percentage of individuals diagnosed with ADHD receiving pharmacological treatment represents an important quality indicator of ADHD care and could be measured at the national level. Untreated ADHD typically affects multiple functional domains of an individual’s well-being, including physical health as well as academic, social, and occupational functioning (18). For example, it may contribute to increased risk of adverse outcomes, including reduced quality of life, emotional and social difficulties, educational underachievement, accidents, criminal behaviour, substance use, and premature death (10). ADHD also imposes significant burdens on families and caregivers, highlighting its broad societal impact (4, 10).

Although effective pharmacological treatments are widely implemented, a multidisciplinary approach combining pharmacotherapy, psychoeducation, behavioural therapy, and psychosocial or psychotherapeutic support can further enhance outcomes and support long-term functioning (4, 10, 11, 19). However, Posner et al. highlighted the fact that non-pharmacological interventions may have demonstrated lower efficacy than previously assumed, while emerging scientific and clinical evidence is beginning to challenge prevailing conceptions of ADHD aetiology (18).

Despite increasing global recognition of the ADHD burden and availability of effective pharmacotherapies, substantial disparities remain in treatment rates across regions, age groups, and health systems (20). Understanding the magnitude and variability in the prevalence of pharmacologically treated ADHD is crucial for identifying treatment gaps and supporting informed healthcare decisions (19).

Most epidemiological studies have focused on diagnosed ADHD prevalence. However, the proportion of individuals who are actually pharmacologically treated may differ substantially, reflecting variations in diagnostic practices, treatment accessibility, clinical guidelines, and cultural attitudes toward medication use (18).

Although some systematic reviews have examined ADHD prevalence or medication use, to the best of our knowledge, no comprehensive meta-analysis has yet quantified the prevalence of pharmacologically treated ADHD across children, adolescents, and adults with a clinically confirmed disorder. Existing evidence remains fragmented, with considerable variability in study design, measurement approaches, and population coverage, and most research to date has focused on specific regions or age groups rather than providing a global perspective.

The present study aims to synthesise available evidence on the prevalence of pharmacologically treated ADHD using a systematic review and meta-analysis. This study will contribute to the existing knowledge of ADHD management. Such data is needed to plan resources for ADHD treatment.

## 2 Methods

This systematic review was conducted in accordance with the Preferred Reporting Items for Systematic Reviews and Meta-Analyses (PRISMA) Statement 2020 (21) and consisted of several phases: identification, screening, eligibility assessment, and inclusion. The protocol for the present systematic review was registered with PROSPERO (CRD42020200220).

### 2.1 Search strategy

A systematic literature search was conducted in PubMed to identify studies reporting the prevalence of pharmacologically treated ADHD across age groups. The search strategy included terms related to “Attention Deficit Hyperactivity Disorder,” and “Prevalence,” and “Pharmacological treatment,” as well as specific medication names such as methylphenidate, amphetamine, lisdexamfetamine, atomoxetine, guanfacine, bupropion, and clonidine. The search covered all records from the database’s inception through October 4, 2024. The complete electronic search strategy is provided in **Supplementary Table 2**. Only studies published in English were included. The search strategy was conducted independently by two researchers (S.Po. and S.Pf), and in cases of disagreement, a third author made the final decision (I.L.). All researchers were qualified in conducting systematic reviews and meta-analyses (especially I.L. & M.S.), which helped to minimize selection bias.

### 2.2 Selection criteria

We included register-based population studies reporting data on individuals diagnosed with ADHD based on clinical diagnosis confirmed by a qualified healthcare professional, according to the International Classification of Diseases (ICD) or the Diagnostic and Statistical Manual of Mental Disorders (DSM) criteria, and documented in official registers or medical records. Only studies providing estimates of ADHD prevalence in general population samples were considered. Studies conducted in non-representative population subgroups (e.g., only males, siblings) or restricted to specific geographic regions (e.g., rural or urban areas with small samples) were excluded. We also excluded studies that did not primarily assess the prevalence of ADHD, studies reporting only lifetime prevalence estimates, and studies including only individuals receiving pharmacological treatment for ADHD. Furthermore, studies in which ADHD diagnoses were not verified through clinical assessment and/or where treatment or diagnostic data were not obtained from official records (e.g., studies relying solely on parental reports of diagnosis or medication use) were excluded. Studies that did not report the proportion of individuals diagnosed with ADHD who received treatment were excluded. Only studies that reported prevalence estimates with confidence intervals (CIs) were included. For studies that did not provide CIs, we attempted to calculate them. Studies for which CIs could not be calculated were excluded.

### 2.3 Data extraction and synthesis

Abstracts were screened against the predefined eligibility criteria. Potentially relevant studies were then assessed through full-text review. Studies meeting all inclusion criteria were included in the final review. Discrepancies between reviewers during both the abstract screening and full-text assessment phases were resolved through discussion and consensus, and when necessary, consultation with an additional reviewer – senior authors (MS, IL). From each eligible study, two independent reviewers extracted data using a standardized data extraction form. References to studies identified in electronic searches were managed in MS Excel^®^. Data collection and extraction were performed independently by two researchers (S.Po. and S.Pf.). The following information was collected: first author and year of publication; year(s) of data collection; reference year of the data presented (in cases with large datasets, data from the most recent year or period were used); study design; country; age range of all participants and relevant subgroups; sex distribution; prevalence of treatment among participants with ADHD; number of ADHD cases; number of pharmacologically treated ADHD cases and diagnostic criteria applied. Detailed characteristics of the included studies, are provided in **Supplementary Table 1**.

Data synthesis was performed by the researchers (S.Po. and S.Pf.), and final verification was carried out by two additional researchers (I.L. and M.S.). Data were synthesized using meta-analytic methods. In the systematic review, data were summarized in tabular form, and relevant outcomes were extracted for meta-analysis. The final synthesis was conducted using the R statistical environment. Age categories were used as subgroups in the meta-analysis utilizing the function *metaprop* within the “meta” package. Due to the expected high heterogeneity, a random-effects model was applied, and the restricted maximum likelihood estimator was used to estimate the between-study variance. The overall prevalence was calculated using a logit transformation to stabilise variances and to account for the bounded nature of proportion data (0% - 100%), thereby improving the statistical properties of the pooled estimates. The confidence interval of the overall prevalence estimate was calculated using the Hartung-Knapp adjustment. Confidence intervals (CIs) for individual study estimates were calculated using exact binomial intervals.

Risk of bias was assessed for all included studies using a previously published methodology, described in detail elsewhere (22). The resulting ratings were used to evaluate study quality but were not incorporated into the meta-analytic weighting procedure.

## 3 Results

### 3.1 Study selection

**Figure 1:**
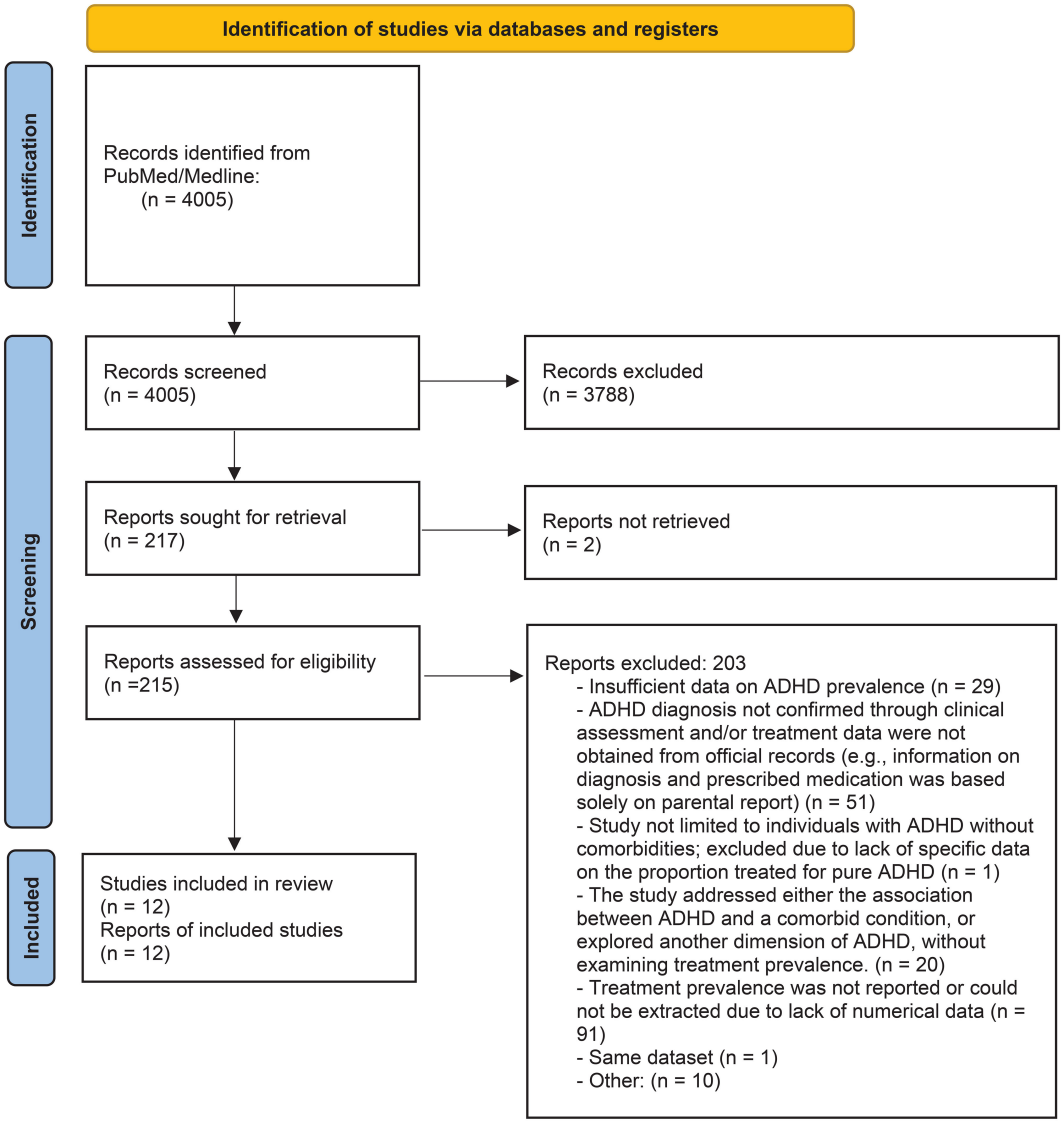
PRISMA flow diagram. Adapted from Page et al., (36), CC BY 4.0, doi: 10.1136/bmj.n71.

The systematic search of the PubMed database initially identified 4,005 records, which two independent reviewers screened based on titles and abstracts. Following this screening, 215 articles were retrieved for full-text assessment, of which 13 studies met all predefined inclusion criteria. The PRISMA flow diagram (**Figure 1**) provides a summary of the study selection process, including the reasons for exclusion at each stage.

### 3.2 Study characteristics

A total of 13 studies conducted across nine countries were included in the systematic review. Studies Song, 2016 and Song, 2018 originated from the same dataset; thus, their estimates were identical for the outcome analyzed. All of the included studies were register-based studies (from electronic health databases). The studies spanned publication years from 2001 to 2024 and covered populations of preschool children (PRE, ≤ 6 years), school children (CHL, 6–12 years), adolescents (ADO, 12–18 years), and adults (ADU ≥ 19 years). A margin of 6 years was allowed for the preschool and school children categories, while an age interval of 11–13 years was allowed for the categories school children and adolescents and an age interval of 17–20 years was allowed for the categories adolescents and adults. Most studies used ICD-based diagnostic criteria (ICD-9 or ICD-10), while one study additionally used DSM-5 criteria. **Table 1** summarizes the key characteristics of the included studies, including cohort time period, period of data collection, country, age category and diagnostic criteria.

**Table 1:** The key characteristics of the included studies in the meta-analysis.

Study	Cohort time period	Year of the collected data	Country	Age	Age category	Diagnostic criteria
Brownell, 2001 (23)	1995 - 1996	1995 - 1996	Canada	≤ 19 years	PRE, CHL, ADO	ICD-9
Chien, 2012 (24)	1996 - 2005	2005	Taiwan	< 18 years	Combined category (PRE-CHL-ADO)	ICD-9
Cho, 2024 (25)	2021	2021	South Korea	6–65 years	CHL, ADO, ADU	ICD-10
Dalsgaard, 2013 (26)	1990 - 2010	1990 - 2010	Denmark	6–17 years	Combined category (CHL-ADO)	ICD-10
Davis, 2021 (27)	2017	2017	US	6–17 years	CHL, ADO	ICD-10
Giacobini, 2018 (28)	2006 - 2011	2006-2011	Sweden	all population	PRE, CHL, ADO, ADU	ICD-10
Giacobini, 2023 (29)	2018 - 2021	2018 - 2021	Sweden	all population	Combined category (PRE-CHL-ADO-ADU)	ICD-10
Raman, 2015 (30)	1994 - 2006	1994 - 2006	UK	3–16 years	PRE, CHL, ADO	ICD, DSM-IV
Song, 2016 (31)*	2007 - 2011	2011	South Korea	1–17 years	PRE, CHL, ADO	ICD-10
Song, 2018 (32)*	2007 - 2011	2011	South Korea	≤ 18 years	Combined category (PRE-CHL-ADO)	ICD-10
Wang, 2016 (33)	2000 - 2009	2000 - 2009	Taiwan	≤ 18 years	Combined category (PRE-CHL-ADO)	ICD-9
Wang, 2017 (34)	2000-2011	2011	Taiwan	≤ 18 years	PRE, CHL, ADO	ICD-9
Winterstein, 2008 (35)	1994 - 2004	2003 - 2004	US	< 20 years	Combined category (PRE-CHL-ADO)	ICD-9

PRE, preschool children; CHL, school children; ADO, adolescents; ADU, adults; ALL, all age groups.

*Studies originate from the same dataset (only the data from Song 2016 were used).

### 3.3 Pooled prevalence

**Figure 2:**
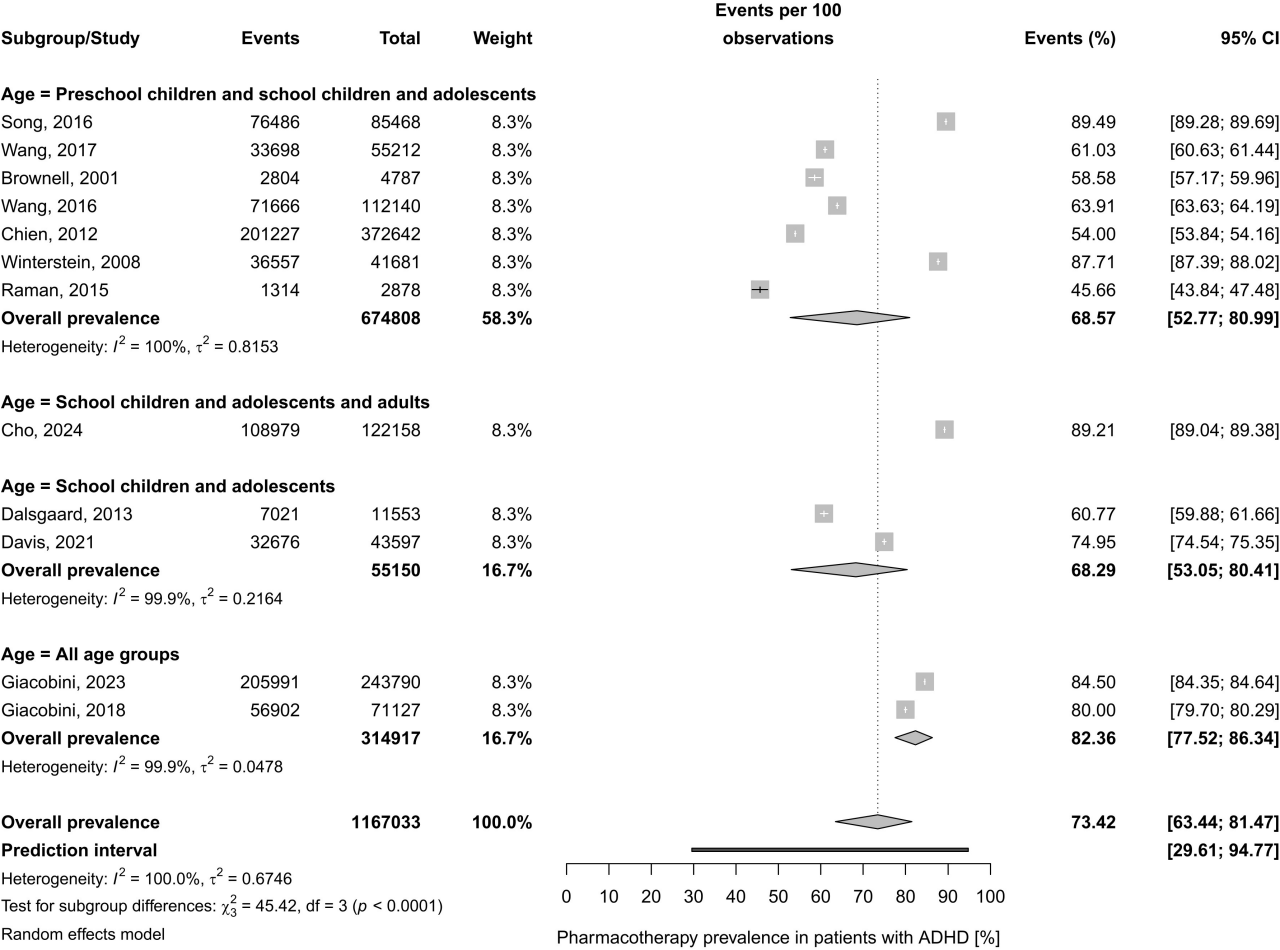
Forest plot of the prevalence of pharmacologically treated ADHD across the studies within the combined age groups as reported in each study. This meta-analysis included all 12 studies.

Studies reporting overall prevalence estimates for pharmacologically treated ADHD (i.e., without stratification by age group) were included in meta-analysis (a total of 12 studies were analysed; **Figure 2**). The pooled prevalence was 73.4% (95% CI: 63.4–81.5), based on a random-effects model. Between-study heterogeneity was very high (I² = 100.0%, τ² = 0.67, p < 0.001), indicating pronounced variability in reported estimates. The highest pooled prevalence was observed in the study of Song et al. (31) that included preschool and school children and adolescents (89.5%, 95% CI: 89.3–89.7). Within each subgroup, heterogeneity remained substantial (I² ≥ 99.9%), reflecting the broad range of population characteristics and study methodologies.

**Figure 3:**
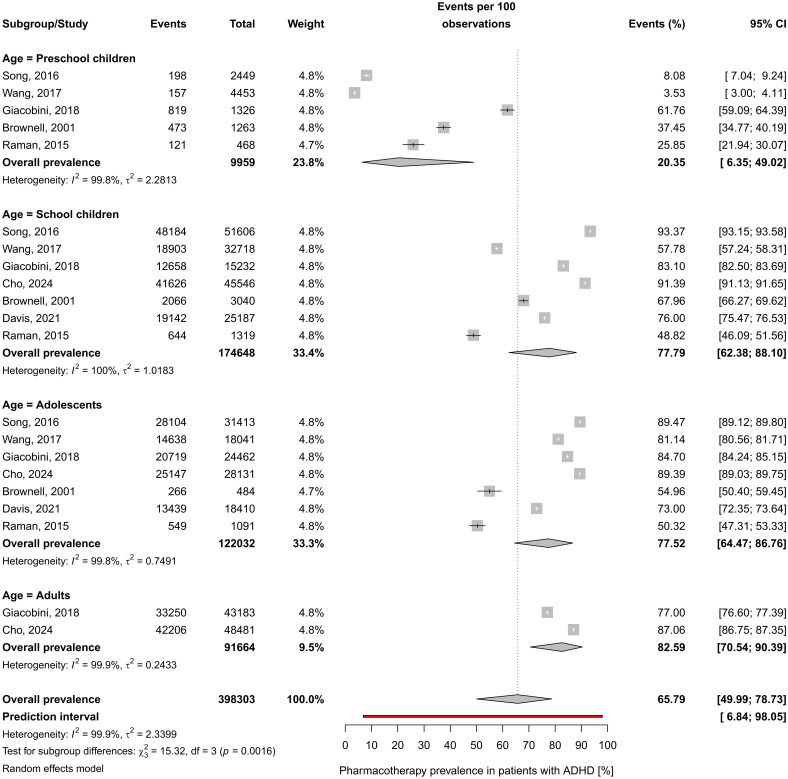
Forest plot of the prevalence of pharmacologically treated ADHD across included studies, stratified by age group. The meta-analysis included 7 studies that reported age-specific prevalence data.

Across studies reporting age-specific data (only 7 studies), the pooled prevalence of pharmacologically treated ADHD was 65.8% (95% CI: 50.0–78.7), with extremely high between-study heterogeneity (I² = 99.9%) (**Figure 3**). Prediction intervals were wide (6.8%–98.1%), indicating substantial variability across populations. Age subgroup analyses showed statistically significant differences (p = 0.0016). Prevalence increased markedly with age, from 20.4% (95% CI: 6.4–49.0) in preschool children to 77.8% (95% CI: 62.4–88.1) in school-aged children and 77.5% (95% CI: 64.5–86.8) in adolescents, reaching 82.6% (95% CI: 70.5–90.1) in adults. Despite stratification, heterogeneity remained extremely high within all age groups. Accordingly, for subgroup adults results are presented as exploratory and should not be interpreted as definitive estimates of age-specific prevalence differences.

**Figure 4:**
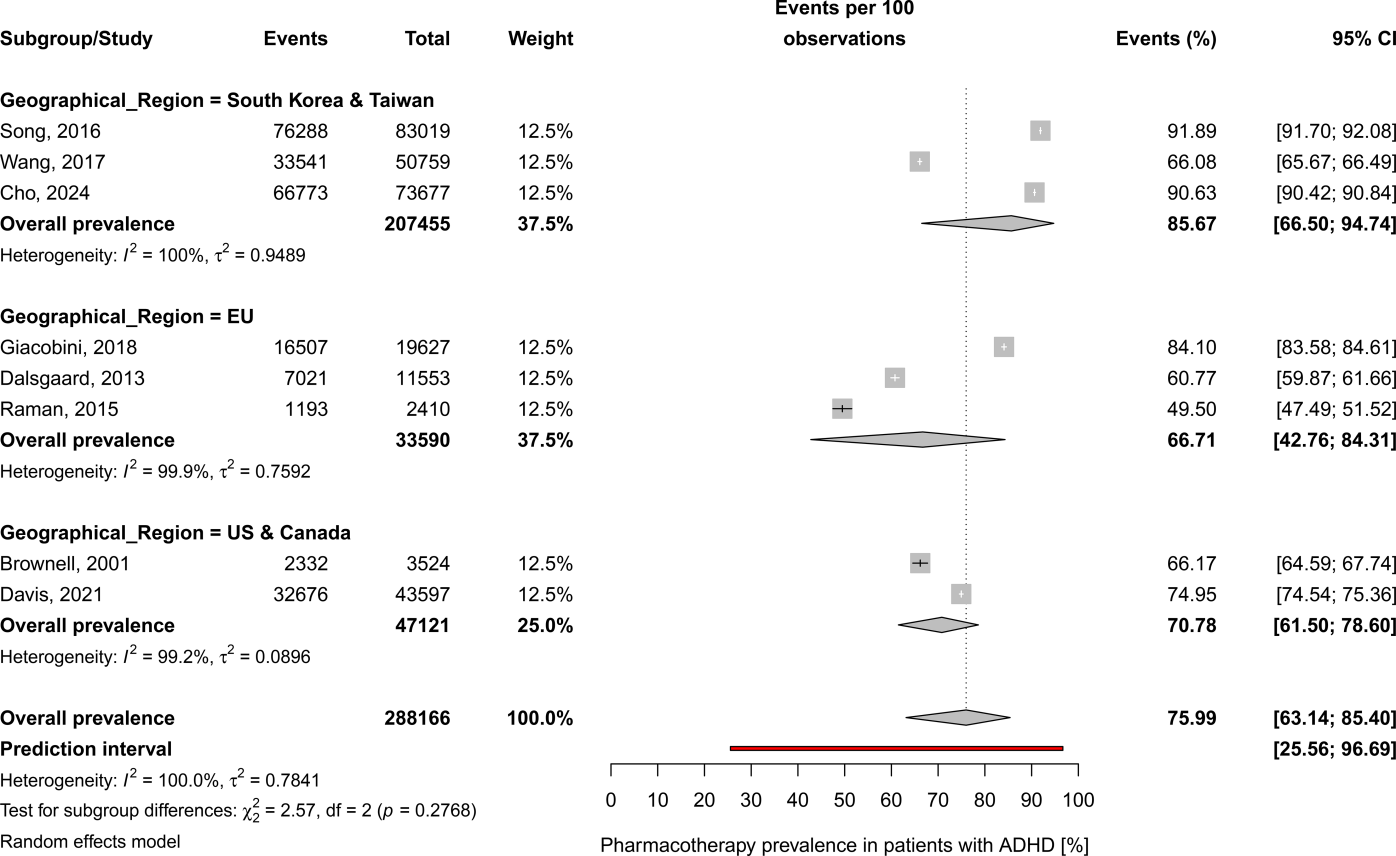
Forest plot of the prevalence of pharmacologically treated ADHD across included studies in the age groups school children and adolescents, stratified by geographical region.

In the subgroup analysis by geographical region the 8 studies reporting data for combined age groups (school-aged children and adolescents) were included. The pooled global prevalence was 76.0% (95% CI: 63.1–85.4), with a wide prediction interval (25.6%–96.7), indicating substantial variability across studies (**Figure 4**). Heterogeneity was very high (I² = 100.0%, τ² = 0.7841). In South Korea and Taiwan the pooled prevalence was 85.7% (95% CI: 66.5–94.7), in the European Union 66.7% (95% CI: 42.8–84.3), and in the United States and Canada 70.8% (95% CI: 61.5–78.6). All subgroups demonstrated considerable heterogeneity. No statistically significant differences were observed between regions (p = 0.2768), suggesting broadly comparable prevalence of pharmacologically treated ADHD across geographical regions in age groups school children and adolescents.

**Figure 5:**
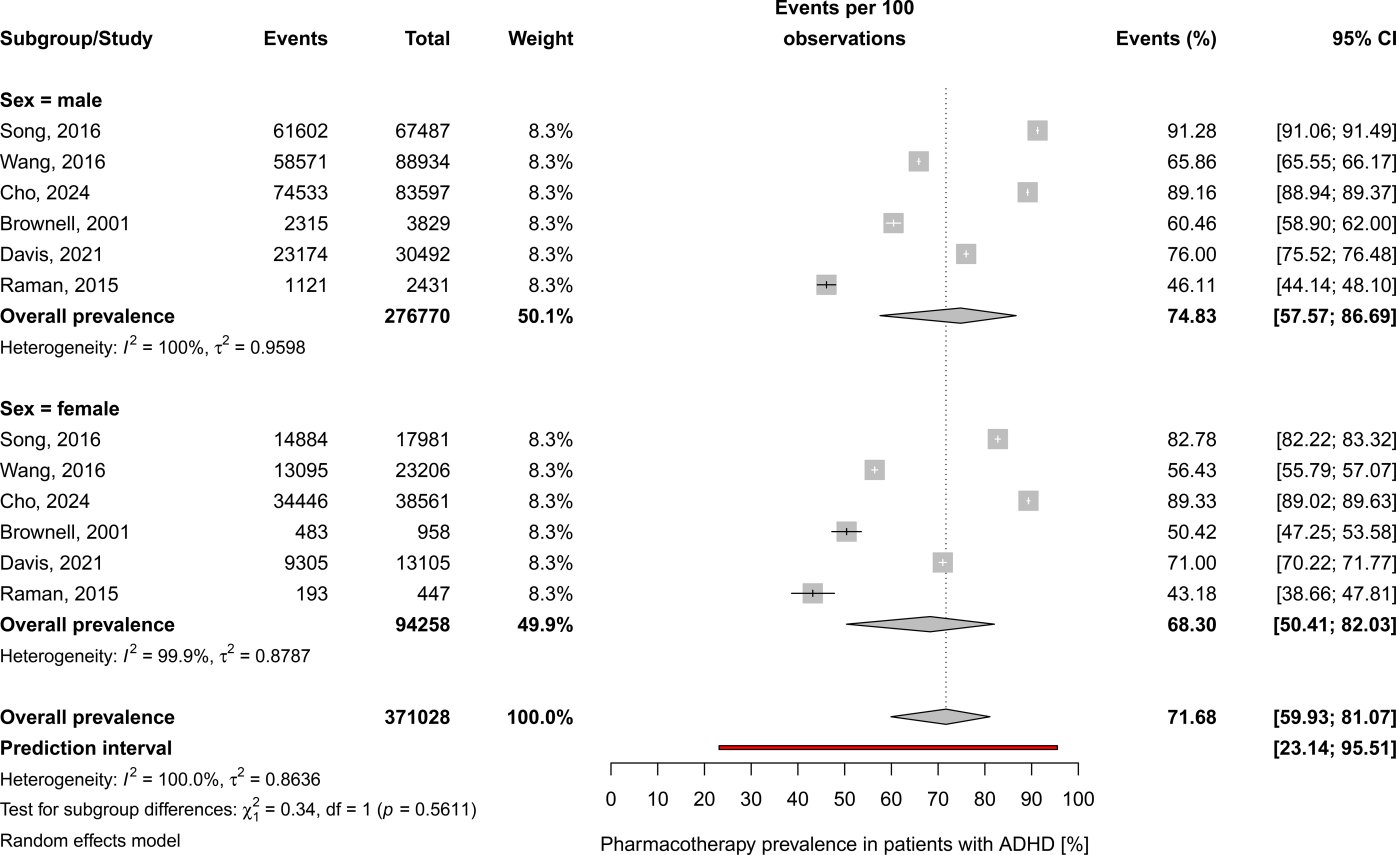
Forest plot of prevalence of pharmacologically treated ADHD across studies, stratified by sex.

Sex-stratified analyses showed a pooled prevalence of 71.7% (95% CI: 59.9–81.1), with no significant difference between males and females (p = 0.56) (**Figure 5**). Point estimates were slightly higher in males than females, although wide confidence intervals and high heterogeneity suggest substantial between-study inconsistency rather than true sex-specific effects. Age groups were not accounted for in this meta-analysis.

Notably, some studies deviated from the general age structure of the included samples, such as Cho (2024) (25), which excluded preschool children but included adults (not represented in other studies), and Davis (2021) (27), which also did not include preschool children. Not all studies included in the systematic review reported sex-disaggregated prevalence of pharmacologically treated ADHD, which prevented quantitative synthesis of sex differences. Nevertheless, several studies provided descriptive information on treatment patterns by sex, allowing a general assessment of male–female differences. Overall, these reports indicate a higher prevalence of pharmacological treatment among males. Giacobini et al. (2023) (29) reported that ADHD treatment prescriptions were more frequent in males (57.2%) than in females (42.8%). Similarly, Giacobini et al. (2018) (28) observed a slightly but significantly higher proportion of treated males. Wang et al. (2017) (34) found that males were more likely to receive medication than females (prevalence ratio = 1.16). Consistently, Chien et al. (2012) (24) identified male sex as a factor associated with higher stimulant use in children with ADHD. Earlier findings by Winterstein et al. (2008) showed that males were more likely than females to receive ADHD medication (4.00% vs. 1.05%), although this difference decreased over time.

### 3.4 Risk of bias

The risk of bias assessment indicated methodological limitations across the included studies, with seven studies rated as having a high risk of bias and the remaining studies classified as having some concerns. Details are listed in **Supplementary Table 3**. Assessments of risk of bias for each included study. These findings should, however, be interpreted in the context of the predominantly register-based design of the included studies. As previously noted, several items within commonly used risk of bias tools are not readily applicable to administrative database studies, highlighting the need for assessment instruments specifically tailored to this type of research (22). Such instruments should also consider design-specific sources of bias, including the use and validation of case definitions, which may influence the accuracy of ADHD identification and treatment ascertainment (22). Consequently, the risk of bias ratings should be interpreted alongside the methodological characteristics of the included studies.

## 4 Discussion

The present meta-analysis provides a comprehensive synthesis of pharmacological treatment prevalence among individuals diagnosed with ADHD across different age groups. The pooled prevalence of 73.4% indicates that the majority of individuals with ADHD receive pharmacological treatment. However, the wide confidence interval and high between-study variability (I² = 100%) suggest substantial variability in treatment practices and population characteristics across studies. While variability across age groups and regions is evident, reflecting differences in healthcare systems and ADHD management practices, the pooled estimate should be interpreted with caution and is not suitable for direct clinical inference, with greater emphasis placed on the observed variation across studies rather than the summary effect alone.

Age-stratified analyses revealed marked differences in treatment prevalence, with the lowest rates observed among preschool children (20.4%) and the highest among adults (82.6%). This pattern likely reflects a combination of clinical caution and regulatory constraints in early childhood, as well as the increasing role of pharmacotherapy as a first-line treatment in older populations. These findings are consistent with current clinical guidelines and prior literature. Pharmacotherapy is generally not recommended for preschool children, which accounts for the low number of treated individuals diagnosed with ADHD in this age group and does not necessarily indicate undertreatment. In contrast, pharmacological treatment is considered first-line therapy for older children and adults with ADHD, and most individuals diagnosed with ADHD in these populations receive medications. These findings are therefore consistent with current guidelines. Further research is needed to clarify the circumstances under which pharmacological treatment may be indicated in preschool children. The highest pooled prevalence among adults indicates that a substantial proportion of individuals with persistent ADHD symptoms continue pharmacological treatment into adulthood. However, the wide confidence intervals, particularly in the adult subgroup, highlight the limited number of studies and variability in adult ADHD diagnosis and treatment practices across countries. In addition, the high proportion of adolescents receiving treatment suggests good medication acceptance in this age group. This may also indicate a good acceptance of pharmacological treatment. Despite stratification, residual heterogeneity remained very high (I² ≈ 100%), which may be attributable to differences in diagnostic criteria (ICD vs. DSM), data sources (prescription records vs. survey data), and national prescribing policies.

Geographic variation in treatment prevalence further underscores the influence of healthcare system characteristics, reimbursement policies, and sociocultural attitudes toward medication use. Higher estimates observed in certain Northern European and East Asian settings contrast with lower estimates in countries such as the UK, Canada, and Taiwan, although these differences should be interpreted with caution given the inconsistency across estimates. Higher treatment rates in Northern European and East Asian (Korean) studies may reflect well-established diagnostic systems, higher treatment accessibility, and greater clinical acceptance of pharmacotherapy for ADHD. Conversely, lower rates reported in Taiwan, Canada, and the UK might stem from more conservative prescribing policies, differences in national treatment guidelines, or cultural attitudes toward medication use in mental health. This could be a point of interest in future studies. Although no statistically significant regional differences were identified, variability across regions—particularly in North America and parts of East Asia—suggests that contextual factors influence treatment patterns despite the overall widespread use of pharmacotherapy. Despite subgrouping, heterogeneity within each age group remained high (I² > 99%), underscoring the influence of contextual factors such as healthcare accessibility, prescribing practices, and national treatment guidelines. Differences in diagnostic systems (ICD vs. DSM) and study periods may also have contributed to this variability. These factors may account for the heterogeneous findings in this meta-analysis. Further exploration using sensitivity analyses could help clarify these findings; however, such analyses were not performed due to the limited number of included studies. Overall, these results highlight a considerable gap between diagnostic recognition and pharmacological management of ADHD in younger populations, while suggesting relatively high treatment coverage among adults. Future studies should aim to disentangle the effects of healthcare policy, comorbidity profiles, and the availability of non-pharmacological treatments on observed treatment prevalence patterns.

Sex-stratified analyses suggest a higher prevalence of pharmacological treatment among males, in line with existing evidence (e.g. Brownell et al. suggested that at every age group, boys are considerably more likely than girls to receive an ADHD diagnosis and be treated with stimulant medication) (23). This pattern may reflect several factors, including higher rates of ADHD diagnosis in boys, differences in symptom presentation (e.g., greater externalizing behaviours in males), and possible gender biases in diagnostic and treatment practices. However, overlapping confidence intervals and more recent data indicate a narrowing of this gap, potentially reflecting improved recognition and management of ADHD in females. Overall, while male predominance in pharmacological treatment remains evident, the narrowing gap across more recent cohorts suggests progress toward more equitable diagnostic and treatment practices. Future studies should aim to disentangle biological, psychosocial, and healthcare-system factors contributing to these patterns and should ensure adequate sex-specific reporting to facilitate cross-national comparisons. Most included studies that reported ADHD prevalence by sex also provided age-stratified data for children, whereas fewer studies reported overall prevalence across all ages. This indicates that age-specific reporting in children is standard, while sex-specific reporting is often secondary. As a result, data on sex differences are mostly limited to the paediatric population, which should be considered when interpreting prevalence estimates and comparing rates between children and adults.

The substantial heterogeneity observed across analyses likely reflects differences in healthcare systems, diagnostic criteria, treatment definitions, study populations, and reporting practices, although the relative contribution of these factors could not be formally quantified. The wide confidence intervals and dispersion in reported prevalence indicate that even within similar healthcare systems, treatment prevalence is influenced by study design, population characteristics, and local prescribing patterns. Furthermore, some of the high national estimates originate from large administrative databases covering insured populations, while lower estimates often derive from smaller or more selective samples. Overall, the findings highlight the strong contextual influence of healthcare systems, reimbursement policies, and sociocultural attitudes on ADHD treatment practices. Future multinational studies with standardized methodologies are needed to better quantify these cross-country differences and to explore whether they reflect true variations in treatment uptake or differences in diagnostic and reporting systems.

In summary, pharmacological treatment is widely utilized in ADHD across the lifespan, but prevalence estimates are strongly influenced by age composition, study design, and contextual factors. Importantly, prevalence estimates of pharmacological treatment in our study do not provide direct information on the quality, appropriateness, or effectiveness of care, which can only be assessed through clinical and real-world studies of treatment outcomes and care delivery. Future research would benefit from standardized, age- and sex-specific reporting, as well as from efforts to better characterize the impact of healthcare systems and sociocultural determinants on treatment patterns.

This study has several limitations that should be acknowledged. The meta-analysis included only register-based studies, which limits the ability to infer causality. Additionally, the literature search was restricted to a single database (PubMed). Although PubMed provides extensive coverage of biomedical and mental health research, relevant studies indexed exclusively in other databases, such as Embase or PsycINFO, may have been missed. Nevertheless, given the stringent inclusion criteria and the detailed study information required for eligibility assessment, we believe that the impact of this limitation on the overall conclusions is likely to be limited. An additional source of heterogeneity may be the variation in how pharmacological treatment was defined across studies, ranging from at least one prescription or prescription claim to more specific criteria based on treatment duration or timing relative to diagnosis. The age-grouping strategy was developed to accommodate heterogeneous reporting across studies and may have introduced some degree of misclassification or imprecision in subgroup estimates. A further limitation is the limited availability of recent studies meeting the eligibility criteria, which may reflect the specificity of the outcome definition rather than the comprehensiveness of the search strategy. A further limitation is the relatively small number of included studies (n = 12), which may have limited the precision and robustness of the meta-analytic estimates. Consequently, several subgroup analyses, including age-, geographic-, and sex-specific comparisons, were based on few studies and may have been underpowered to detect meaningful differences between groups. Different populations were included, resulting in a limited number of studies for some subgroups and limiting the feasibility of sensitivity analyses stratified by population characteristics. Confidence intervals were often wide, reflecting high variability within studies. Furthermore, studies from certain regions of the world were excluded, limiting the generalizability of the findings. Differences in diagnostic criteria across studies may have contributed to observed heterogeneity and should be considered in the interpretation of pooled estimates. Furthermore, most included studies were registry-based, for which currently available risk of bias tools may not fully capture design-specific sources of bias, potentially affecting the interpretation of study quality assessments. Finally, although a formal risk of bias assessment was performed, the findings were not stratified or weighted according to risk of bias. Therefore, the extent to which study quality may have influenced the pooled estimates remains uncertain.

Our meta-analysis did not examine overall ADHD medication consumption, prescription rates, or medication use in the general population. Rather, the objective was to estimate the pooled prevalence of pharmacologically treated individuals among those with clinically diagnosed ADHD - as defined by ICD or DSM criteria. Consequently, studies were eligible only if they provided information on both the prevalence of clinically diagnosed ADHD and the proportion of individuals with clinically diagnosed ADHD receiving pharmacological treatment. Studies reporting medication utilization or prescription rates alone were not eligible for inclusion because they do not allow estimation of treatment prevalence among individuals with ADHD. However, increasing prescription rates do not necessarily translate into a higher proportion of individuals with ADHD being treated, as trends in diagnosis, case ascertainment, healthcare access, and population denominators may also influence these estimates.

Studies reporting medication utilization or prescription rates alone were excluded because they do not permit estimation of treatment prevalence among individuals with ADHD. Moreover, increases in prescription rates do not necessarily correspond to a higher proportion of individuals with ADHD receiving treatment, as such trends may also reflect changes in diagnostic practices, case ascertainment, healthcare access, or underlying population denominators. Therefore, our findings should not be interpreted as estimates of national medication consumption or prescribing intensity, but rather as estimates of treatment coverage among individuals with clinically diagnosed ADHD.

The findings of this meta-analysis indicate substantial global variation in the prevalence of ADHD pharmacotherapy. Overall, a relatively high proportion of individuals diagnosed with ADHD receive pharmacological treatment, reflecting a positive trend. However, a notable percentage of preschool-aged children are also being treated, which warrants further investigation in future studies. The substantial heterogeneity observed across analyses likely reflects differences in healthcare systems, diagnostic criteria, treatment definitions, study populations, and reporting practices, although the relative contribution of these factors could not be formally quantified. This between-study variation highlights the importance of contextual factors when interpreting treatment prevalence estimates and may suggest differences in access to, or delivery of, pharmacological treatment across settings. Greater standardization in the definition and reporting of treatment prevalence would improve comparability across studies.

## Data Availability

The original contributions presented in the study are included in the article/**Supplementary Material**. Further inquiries can be directed to the corresponding author.

## Supplementary material

Supplementary Table 1 Data Extraction from Included Studies.

Supplementary Table 2 The complete electronic search strategy. PRISMA Checklist.

Supplementary Table 3 Assessments of risk of bias for each included study.

Supplementary Table 4 PRISMA Checklist.
