# Supplementary material for: Prevalence of pharmacologically treated attention deficit hyperactivity disorder in children, adolescents, and adults: systematic review and meta-analysis
Source: Front Psychiatry. 2026 Jul 10;17:1854611. doi: 10.3389/fpsyt.2026.1854611 (PMC13397222; doi:10.3389/fpsyt.2026.1854611)
Supplement: Supplementary Table 2 — The complete electronic search strategy. PRISMA Checklist. [file Table2.docx]

Supplementary Material

# Supplementary Data

Supplementary Table S1. Full electronic search strategy used in PubMed.

| ("attention deficit disorder with hyperactivity"[MeSH Terms] OR "attention deficit disorder with hyperactivity"[Title/Abstract] OR "attention deficit disorders with hyperactivity"[Title/Abstract] OR "attention deficit hyperactivity disorder*"[Title/Abstract] OR "attention deficit disorder*"[Title/Abstract] OR "ADHD"[Title/Abstract] OR "ADDH"[Title/Abstract] OR "minimal brain dysfunction*"[Title/Abstract] OR "minimal cerebral dysfunction*"[Title/Abstract] OR "hyperkinetic syndrome*"[Title/Abstract] OR "hyperkinetic disorder"[Title/Abstract] OR "hyperactive impulsiv*"[Title/Abstract] OR "hyperkines*"[Title/Abstract])  AND  ("epidemiology"[MeSH Terms] OR "prevalence"[MeSH Terms] OR "epidemio*"[Title/Abstract] OR "prevalence*"[Title/Abstract] OR "Rate"[Title/Abstract] OR "point estimate"[Title/Abstract])  AND  ("Methylphenidate"[Title/Abstract] OR "Methylphenidate"[MeSH Terms] OR "atomoxetine"[Title/Abstract] OR "Atomoxetine Hydrochloride"[MeSH Terms] OR "bupropion"[Title/Abstract] OR "buproprion"[Title/Abstract] OR "bupropion"[MeSH Terms] OR "guanfacine"[Title/Abstract] OR "clonidine"[Title/Abstract] OR "clonidine"[MeSH Terms] OR "mixed amphetamine salts"[Title/Abstract] OR "lisdexamphetamine"[Title/Abstract] OR "Lisdexamfetamine Dimesylate"[MeSH Terms] OR "amfetamine"[Title/Abstract] OR "amphetamine"[Title/Abstract] OR "Amphetamines"[MeSH Terms] OR "stimulant*"[Title/Abstract] OR "Central Nervous System Stimulants"[MeSH Terms] OR "Pharmaceutical Preparations"[MeSH Terms] OR "medication*"[Title/Abstract] OR "Drug Therapy"[MeSH Terms] OR "pharmacotherapy"[Title/Abstract] OR "pharmacological treatment"[Title/Abstract] OR "pharmacolog*"[Title/Abstract] OR "prescription*"[Title/Abstract] OR "Prescriptions"[MeSH Terms] OR "treat*"[Title/Abstract]) |
| --- |
