# Supplementary material for: Prevalence of pharmacologically treated attention deficit hyperactivity disorder in children, adolescents, and adults: systematic review and meta-analysis
Source: Front Psychiatry. 2026 Jul 10;17:1854611. doi: 10.3389/fpsyt.2026.1854611 (PMC13397222; doi:10.3389/fpsyt.2026.1854611)
Supplement: Supplementary Table 3 — Assessments of risk of bias for each included study. [file Table3.docx]

Supplementary Material

# Supplementary Data

**Supplementary Table S3. Assessments of risk of bias for each included study.**

| **Lead author** | **D1. Risk of bias due to sample selection** | **D2. Risk of bias due to improper measurement of the condition** | **D4. Risk of Bias in Statistical Analysis** | **D3. Risk of bias due to non-response** | **Overall RoB** |
| --- | --- | --- | --- | --- | --- |
| Brownell, 2001 (1) | Low | Some concerns | Low | / | Some concerns |
| Davis, 2021 (2) | Low | Some concerns | Low | / | Some concerns |
| Giacobini, 2018 (3) | High | Some concerns | Low | / | High RoB |
| Giacobini, 2023 (4) | High | Some concerns | Low | / | High RoB |
| Wang, 2017 (5) | Low | Some concerns | Low | / | Some concerns |
| Song, 2016 (6) | High | Some concerns | Low | / | High RoB |
| Cho, 2024 (7) | Low | Some concerns | Low | / | Some concerns |
| Wang, 2016 (8) | High | Some concerns | Low | / | High RoB |
| Chien, 2012 (9) | High | Some concerns | Low | / | High RoB |
| Winterstein, 2008 (10) | High | Some concerns | Low | / | High RoB |
| Dalsgaard, 2013 (11) | High | Some concerns | Low | / | High RoB |
| Raman, 2015 (12) | Low | Some concerns | Low | / | Some concerns |
| Song, 2018 (13) | Low | Some concerns | Low | / | Some concerns |

1. Brownell MD, Yogendran MS. Attention-deficit hyperactivity disorder in Manitoba children: medical diagnosis and psychostimulant treatment rates. *Can J Psychiatry* (2001) 46:264–272. doi: 10.1177/070674370104600307

2. Davis DW, Jawad K, Feygin Y, Creel L, Kong M, Sun J, Lohr WD, Williams PG, Le J, Jones VF, et al. Disparities in ADHD Diagnosis and Treatment by Race/Ethnicity in Youth Receiving Kentucky Medicaid in 2017. *Ethn Dis* (2021) 31:67–76. doi: 10.18865/ed.31.1.67

3. Giacobini M, Medin E, Ahnemark E, Russo LJ, Carlqvist P. Prevalence, Patient Characteristics, and Pharmacological Treatment of Children, Adolescents, and Adults Diagnosed With ADHD in Sweden. *J Atten Disord* (2018) 22:3–13. doi: 10.1177/1087054714554617

4. Giacobini M, Ahnemark E, Medin E, Freilich J, Andersson M, Ma Y, Ginsberg Y. Epidemiology, Treatment Patterns, Comorbidities, and Concomitant Medication in Patients with ADHD in Sweden: A Registry-Based Study (2018-2021). *J Atten Disord* (2023) 27:1309–1321. doi: 10.1177/10870547231177221

5. Wang T, Liu K, Li Z, Xu Y, Liu Y, Shi W, Chen L. Prevalence of attention deficit/hyperactivity disorder among children and adolescents in China: a systematic review and meta-analysis. *BMC Psychiatry* (2017) 17:32. doi: 10.1186/s12888-016-1187-9

6. Song I, Shin J-Y. Prescribing patterns for attention deficit hyperactivity disorder medications among children and adolescents in Korea, 2007-2011. *Epidemiol Health* (2016) 38:e2016045. doi: 10.4178/epih.e2016045

7. Cho Y, Kim A-Y, Lee S, Lee H. Recent updates on treatment patterns in patients with treated attention-deficit/hyperactivity disorders from a nationwide real-world database in South Korea. *Int Clin Psychopharmacol* (2024) 39:240–249. doi: 10.1097/YIC.0000000000000549

8. Wang L-J, Yang K-C, Lee S-Y, Yang C-J, Huang T-S, Lee T-L, Yuan S-S, Shyu Y-C. Initiation and Persistence of Pharmacotherapy for Youths with Attention Deficit Hyperactivity Disorder in Taiwan. *PLoS One* (2016) 11:e0161061. doi: 10.1371/journal.pone.0161061

9. Chien I-C, Lin C-H, Chou Y-J, Chou P. Prevalence, incidence, and stimulant use of attention-deficit hyperactivity disorder in Taiwan, 1996-2005: a national population-based study. *Soc Psychiatry Psychiatr Epidemiol* (2012) 47:1885–1890. doi: 10.1007/s00127-012-0501-1

10. Winterstein AG, Gerhard T, Shuster J, Zito J, Johnson M, Liu H, Saidi A. Utilization of pharmacologic treatment in youths with attention deficit/hyperactivity disorder in Medicaid database. *Ann Pharmacother* (2008) 42:24–31. doi: 10.1345/aph.1K143

11. Dalsgaard S, Nielsen HS, Simonsen M. Five-fold increase in national prevalence rates of attention-deficit/hyperactivity disorder medications for children and adolescents with autism spectrum disorder, attention-deficit/hyperactivity disorder, and other psychiatric disorders: a Danish register-based study. *J Child Adolesc Psychopharmacol* (2013) 23:432–439. doi: 10.1089/cap.2012.0111

12. Raman SR, Marshall SW, Gaynes BN, Haynes K, Naftel AJ, Stürmer T. An observational study of pharmacological treatment in primary care of children with ADHD in the United kingdom. *Psychiatr Serv* (2015) 66:617–624. doi: 10.1176/appi.ps.201300148

13. Song I, Lee MS, Lee E-K, Shin J-Y. Patient and provider characteristics related with prescribing of ADHD medication: Nationwide health insurance claims database study in Korea. *Asia Pac Psychiatry* (2018) 10: doi: 10.1111/appy.12289
